# *Detarium microcarpum, Guiera senegalensis, and Cassia siamea* Induce Apoptosis and Cell Cycle Arrest and Inhibit Metastasis on MCF7 Breast Cancer Cells

**DOI:** 10.1155/2019/6104574

**Published:** 2019-05-23

**Authors:** Ismail Abiola Adebayo, Haladu Ali Gagman, Wasiu Gbolahan Balogun, Mowaffaq Adam Ahmed Adam, Rafedah Abas, Khalid Rehman Hakeem, Nik Ahmad Irwan Izzauddin Bin Nik Him, Muhammad Razip Bin Samian, Hasni Arsad

**Affiliations:** ^1^Integrative Medicine Cluster, Advanced Medical and Dental Institute, Universiti Sains Malaysia, Bertam, 13200 Kepala Batas, Pulau Pinang, Malaysia; ^2^School of Biological Sciences, Universiti Sains Malaysia, 11800 Penang, Malaysia; ^3^Department of Biological Sciences, Faculty of Sciences, Bauchi State University Gadau, 751 Itas Gadau, Nigeria; ^4^Infectomics Cluster, Advanced Medical and Dental Institute, Universiti Sains Malaysia, Penang, Malaysia; ^5^Centralized Research Labs (CRL), Advanced Medical and Dental Institute USM, Bertam, 13200 Kepala Batas, Penang, Malaysia; ^6^Department of Biological Science, Faculty of Science, King Abdulaziz University, PO Box 80203, Jeddah, Saudi Arabia

## Abstract

Despite the availability of anticancer drugs, breast cancer remains the most death-causing tumor-related disease in women. Hence, there is a need for discovery and development of efficient alternative drugs, and sources such as plants need to be explored. In this study, antioxidant capacities and inhibitory effects against MCF7 cells of the extracts of stem bark of three Nigerian medicinal plants (*Detarium microcarpum*,* Guiera senegalensis, *and* Cassia siamea*) were investigated. The* D. microcarpum *extracts had the highest antioxidant and antiproliferative effects, followed by that of* G. senegalensis*, and the* C. siamea *extracts had minimal effects. The IC_50_ values of the methanol and aqueous extracts from the three plants that inhibited the proliferation of MCF7 cells ranged from 78–> 500 *μ*g/ml. Moreover, all the plant extracts but the aqueous extract of* Cassia siamea *exhibited antimetastatic action and induced apoptosis and cell cycle arrest in MCF7 cells. Liquid chromatography/time-of-flight/mass spectrometry profiling revealed that the five potent extracts contain many phenols and omega-6 fatty acids, and some of the identified compounds (isorhamnetin, eupatorin, alpinumisoflavone, procyanidin B3, syringin, and gallic acid) have been reported to have antiproliferative effects on cancer cells. Hence, the stem bark of these plants could be potential sources of antibreast cancer agents.

## 1. Introduction

Breast cancer is the most death-causing tumor-related disease in females [1]. About 70% of breast tumors are estrogen receptor positive, and the percentage is higher among older females [2]. The persistence of this major health problem is due to many factors, including the growing resistance of breast cancer cells to conventional drugs, the highly metastatic nature of the tumors, and the high cost of anticancer drugs, which makes them inaccessible to poor patients. Hence, herbal medicines are being explored as alternative sources of breast cancer chemotherapy agents [1, 3–5].

The selected plants for this study have ethnopharmacological relevance and they are commonly used in Africa for the purpose.* Detarium microcarpum*,* Cassia siamea*, and* Guiera senegalensis *are popular medicinal plants in West Africa. The bark of these plants is used as medicine to treat diseases in Nigeria and other West African countries.* D. microcarpum *is a perennial tree that usually reaches 4–5 m in height. It is widely grown in the northern part of Nigeria and other Sub-Saharan African countries [6]. In Folklore medicine,* D. microcarpum *is considered to be a potent medicinal herb, and it is traditionally used to cure and prevent many diseases, including oxidative stress-related ailments such as cancer.* D. microcarpum *has been scientifically shown to have antimicrobial, hepatoprotective, cytotoxic, and antidiabetic effects [6–8].* G. senegalensis *belongs to the family Combretaceae. It is widely planted in the savannah region of Africa. Traditionally,* G. senegalensis *is a medicinal herb that is used to cure many diseases and ailments, including wound infections, jaundice, diabetes, arthritis, fever, diarrhea, gastrointestinal and respiratory diseases, and cancer [9–11].* C. siamea *is also a plant that has ethnopharmacological importance. It is traditionally employed in tropical countries, including Nigeria, to prevent and treat diseases such as diabetes, insomnia, high blood pressure, and constipation [12]. It was recently reported that the ethanolic extracts of* C. siamea *possess significant antioxidant and antibacterial properties [12].

Therefore, in this study, the antioxidant activities and inhibitory effects of extracts of* D. microcarpum*,* C. siamea*, and* G. senegalensis *stem bark against estrogen responsive breast cancer (MCF7) cells were investigated.

## 2. Methodology

### 2.1. Plant Collection and Authentication

The bark samples were collected from the stems of plants that were harvested from the wide forest of Azare, Katagum Local Government Area, Bauchi State, Nigeria between October and December 2015. The plants were authenticated at the Biological Science Department of Ahmadu Bello University, Zaria, Nigeria. Voucher numbers were allocated to the plant samples as follows:* Detarium microcarpum *(3105),* Cassia siamea *(900078), and* Guiera senegalensis *(900103), and plant samples were deposited at the University Herbarium.

### 2.2. Preparation of the Plant Extracts

Extraction of phytochemicals from the bark samples was performed using water and methanol solvents. The stem bark was peeled off and washed thoroughly. It was air-dried under shade at room temperature for 3 weeks and later crushed to powder. For extraction, 50 g of the powder was added to 250 ml of distilled water or methanol and the mixture was vigorously mixed.

The mixture was left for 5 days under constant agitation. The extraction process was repeated one more time with the same powder sample. The liquid extract was filtered from the mixture using Whatman No. 1 filter paper. The filtrate was oven-dried at 45°C, and the dry extract was stored at 4°C until further use. The extracts were labelled as follows:* Detarium microcarpum *methanol extract (DMME),* Cassia siamea *methanol extract (CSME),* Guiera senegalensis *methanol extract (GSME),* Detarium microcarpum *aqueous extract (DMAE),* Cassia siamea *aqueous extract (CSAE), and* Guiera senegalensis *aqueous extract (GSAE).

### 2.3. ABTS Radical Scavenging Activity

Antioxidant capacities of the methanol and aqueous extracts were determined using the ABTS test [13, 14]. Prior to the assay, 7 mM of ABTS was reacted with 2.45 mM of potassium persulfate in equal volumes for 16–18 hours to produce ABTS^·+^ solution. The ABTS^·+^ solution was then diluted with 50% methanol to make working solution and the absorbance of the ABTS^·+^ working solution was adjusted to an approximate value of 1 at 754 nm. Twenty microliters of five concentrations (3.91, 7.81, 15.63, and 31.25 *μ*g/ml final concentrations for* D. Microcarpum *and* G. senegalensis *extracts; 31.25, 62.5, 125, 250, and 500 *μ*g/ml final concentrations for* C. siamea *extracts) was separately mixed with 180 *μ*l of the ABTS^·+^ working solution in a 96-well plate. The mixtures were incubated in the dark for 6 minutes, after which their absorbances were read at 754 nm. Percentage ABTS radical scavenging activity was calculated for each concentration of the samples using the following formula:

(1) Percentage  radical  scavenging  activity%=AB−AS÷AB×100

where AB is absorbance of the blank (0 *μ*g/ml of sample) and AS is absorbance of the sample.

A regression curve was obtained for each sample by plotting the value of percentage radical scavenging activity against its corresponding concentration of the samples. IC_50_ was then extrapolated from the curve.

### 2.4. DPPH Radical Scavenging Activity

The DPPH antioxidant test was also used to determine the ability of the samples to scavenge free radicals. The test was performed according to the method of Adebayo, Arsad [15] with slight modifications. One hundred and eighty microliters of 0.2 mM DPPH methanol solution was mixed with 12 *μ*l of different concentrations of the samples (2.44, 4.88, 9.77, 19.53, and 39.06 *μ*g/ml final concentrations for* D. microcarpum *and* G. senegalensis *extracts; 19.53, 78.13, 156.25, 312.5, and 625 *μ*g/ml final concentrations for* C. siamea* extracts). The mixtures were incubated in the dark for 20 minutes at room temperature. Their absorbances were read at 517 nm. Percentage DPPH radical scavenging activities of the samples were determined following the procedure described above for the ABTS test, and regression curves were obtained and IC_50_ values for each sample were extrapolated.

### 2.5. Cell Culture System

MCF7 breast cancer cells (American Type Culture Collection, Manassas, VA, USA) were cultured and maintained in RPMI 1649 medium supplemented with 10% foetal bovine serum and 1% antibiotics penicillin/ streptomycin (pen/strep). The cells were subcultured after 5–6 days when they reached > 80% confluence.

### 2.6. Presto Blue Cell Viability Assay

MCF7 cells were harvested from the exponentially growing culture. Ninety microliters of 5000 cells/ml was seeded in each well of 96-well plates for 24 hours. The medium was discarded and replaced with extract-diluted medium of different concentrations (15.625, 31.25, 62.5, 125, 250, and 500 *μ*g/ml) of the samples or dimethyl sulfoxide (DMSO, vehicle). The assays were incubated for 72 hours, after which 10 *μ*l of Presto Blue resazurin dye (Thermo Fisher Scientific, Waltham, MA, USA) was added to each well according to the manufacturer's protocol. The stained cells were incubated for 10 minutes at 37°C. Following incubation, absorbances were read at about 570 nm (excitation) and 600 nm (emission).

Percentage cell viability was calculated based on the manufacturer's instructions.

### 2.7. Wound Healing Assay

The wound healing assay was performed to investigate the antimetastatic effect of the active extracts on MCF7 cell progression. The experiment was performed according to the method of Baharuddin, Satar [16]. MCF7 cells were grown to confluence in wells of 12-well plate, and a sterile 200 *μ*l pipette tip was used to create a single scratch at the center of each well. The medium was removed and the scratched cell debris was washed away with phosphate buffered saline. Then, DMSO containing medium (control) or extract containing medium was added to the well. Images of the treated cells were captured at three different time points (0, 12, and 18 hours) using an inverted light microscope. The areas of the scratches before and after treatment were estimated using Image J software. Percentage of wound healed was calculated using the following formula:

(2) %  wound  healed=Initial  scratch  area−Final  scratch  areaInitial  scratch  area×100

### 2.8. Cell Cycle Analysis

An optimized number of 50,000 MCF7 cells were seeded into each well of a 6-well plate for 24 hours. The medium was discarded and replaced with DMSO (vehicle), puromycin (positive control), or plant extract-diluted medium using IC_50_ and 2IC_50_ concentrations. The cells were further incubated for 72 hours. The cell cycle analysis kit (Invitrogen, Thermo Fisher Scientific, Carlsbad, CA, USA) used was applied as recommended by the manufacturer. Briefly, the cells were harvested using trypsin and fixed with 70% ethanol for about 1 hour at 4°C. Then, 50 *μ*l of propidium iodide (PI) stain was added to the cells (≤ 1 × 10^6^ cells), and the solution was vortexed. The stained cells were incubated in the dark at room temperature for 15–30 minutes. Lastly, the cells were analyzed by flow cytometer.

### 2.9. Apoptosis Assay

The apoptosis assay was performed using Annexin V/PI reagents according to manufacturer's instruction (BD Biosciences, Qume Drive, San Jose, CA). Following seeding of MCF7 cells and subsequent treatment as described above for the cell cycle assay, the cells were harvested using 0.005% trypsin solution. Both the medium and the cells were collected and the cells were centrifuged. The cells were resuspended in 1 ml 1× binding buffer (at the rate of 1 × 10^6^ cells/ml). Next, 100 *μ*l of the cell solution was transferred to a 5 ml tube, and 5 *μ*l of FITC Annexin V and 5 *μ*l of PI was added to the solution. The cells were gently vortexed and incubated for 15 minutes in the dark at room temperature. Finally, 400 *μ*l of 1× binding buffer was added to the sample, and the sample was immediately analyzed by flow cytometer.

### 2.10. LC/TOF/MS

The metabolites of the medicinal plant extracts were profiled by LC/TOF/MS using a Waters Alliance 2795LC device (Milford Massachusetts, USA). An aliquot of 1 mg/ml (w/v) of each plant extract was used. Five microliters of the extract was injected into the LC instrument, which was coupled with a Waters Alliance 2795 column. The mobile phases were 0.1% formic acid in water (solvent A) and 10% acetonitrile in water (solvent B) at a flow rate of 0.7 ml/min. The LC conditions were 90% A and 10% B from 0 to 2 minutes and 10 to 40% B from 2 to 15 min. This condition was maintained for 2 minutes, which marked the end of the run. The MS analysis was performed using an LCT Premier XE #KE376 instrument by electrospray ionization (ESI) in negative mode. MS data were acquired within the m/z range of 100 to 1000. The acquired m/z values were calibrated by leucine encephalin (molecular mass = 555.632 g/mol). The voltage of the capillary was 3 kV and its temperature was 120°C. The acquired data (mz values and spectra) were processed for identification of probable compounds using an online database (https://metlin.scripps.edu/landing_page.php?pgcontent=mainPage) [17].

### 2.11. Statistical Analysis

Numerical values are expressed as mean ± standard error. The level of significance (p value) of the data was determined using one-way ANOVA. Data with p values that are less than 0.01 and 0.05 are considered significant.

## 3. Results

### 3.1. Antioxidant Capacities of the Plant Extracts

The antioxidant capacities of the plant extracts were quantitatively determined using DPPH and ABTS radical scavenging tests because the ability to quench or suppress reactive oxygen species and free radicals in living organisms is crucial for treatment of many diseases such as cancer, hypertension, and other oxidative stress-related illnesses. The results showed that the plant extracts have significant antioxidant capacities (supplementary data (available here)). In ascending order, the IC_50_ values of the extracts for the ABTS test were 7.67 ± 0.74 *μ*g/ml (DMAE) < 8.97 ± 0.15 *μ*g/ml (DMME) < 11.77 ± 0.37 *μ*g/ml (GSAE) < 12.75 ± 0.38 *μ*g/ml (GSME) < 115 ± 1.53 *μ*g/ml (CSAE) < 160 ± 3.82 *μ*g/ml (CSME). In ascending order, the IC_50_ values of the extracts for the DPPH test were 12.93 ± 0.13 *μ*g/ml (DMAE) < 16.2 ± 0.31 *μ*g/ml (DMME) < 18.83 ± 0.67 *μ*g/ml (GSAE) < 19.07 ± 0.98 *μ*g/ml (GSME) < 232 ± 6.03 *μ*g/ml (CSME) < 464 ± 72.15 *μ*g/ml (CSAE). DMAE had the lowest IC_50_, hence the highest antioxidant capacity, whereas CSME and CSAE had the highest IC_50_ values in both tests. GSAE and GSME had almost equal antioxidant capacities. The results of the DPPH and ABTS tests were highly correlated (R^2^ = 0.7), which mean they were reliable and significant. Therefore, the results showed that all six extracts had significant and dose-dependent antioxidant capacities.

### 3.2. Antiproliferative Effects of the Plant Extracts on MCF7 Cell Growth

The antiproliferative effects of the plant extracts on MCF7 cell growth were determined using the Presto Blue cell viability assay, and Table 1 shows the results. DMAE had the highest inhibitory effect with the lowest IC_50_ value of 78 *μ*g/ml. Other extracts showed remarkable inhibitory effects as well, with IC_50_ values of 130 *μ*g/ml (DMME), 141 *μ*g/ml (GSAE), 182 *μ*g/ml (GSME), and 129 *μ*g/ml (CSME). However, CSAE had little effect on the proliferation of MCF7 cells, as more than half of the cell population treated with 500 *μ*g/ml of the extract was actively viable after 72 hours.

### 3.3. Antimigration Effects of the Five Potent Extracts on MCF7 Progression

**Figure 1:**
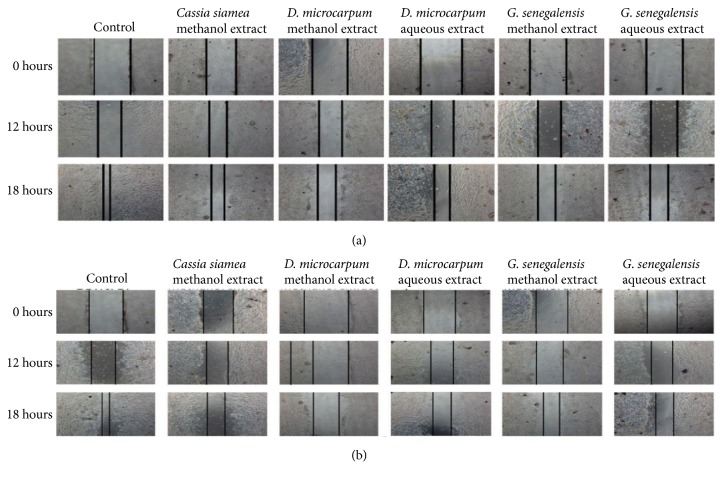
*Effect of the plant extract on cell migration.* A wound healing assay was used to determine the effect of the potent extracts on the migration of MCF7 cells. The figure displays the artificial wounds in the treated and untreated MCF7 cells after treatment with IC50 (a) and 2IC50 (b) concentrations of the plant extracts.* Detarium microcarpum *methanol extract (DMME),* Cassia siamea *methanol extract (CSME),* Guiera senegalensis *methanol extract (GSME),* Detarium microcarpum *aqueous extract (DMAE), and* Guiera senegalensis *aqueous extract (GSAE).

**Figure 2:**
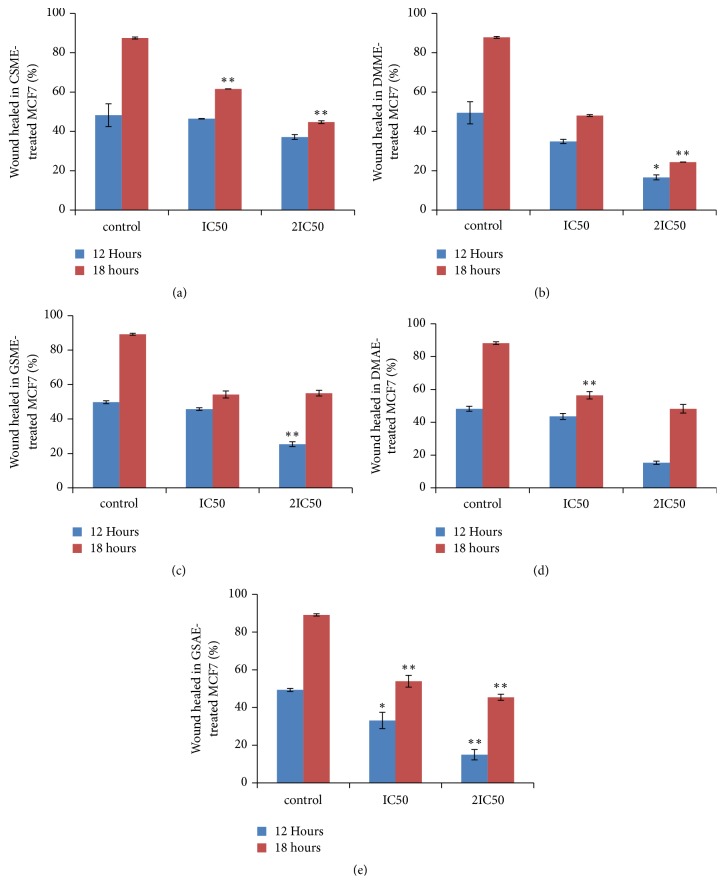
*Percentages of wound healed after treatment of viable MCF7 cells with the plant extracts for 72 hours. *The figure displays the percentages area of wounds of the treated MCF7 cells relative to the untreated MCF7 cells after treatment with IC50 and 2IC50 concentrations of the plant extracts;* Cassia siamea *methanol extract (CSME) (a),* Detarium microcarpum *methanol extract (DMME) (b),* Guiera senegalensis *methanol extract (GSME) (c),* Detarium microcarpum *aqueous extract (DMAE) (d), and* Guiera senegalensis *aqueous extract (GSAE) (e). Values that are marked with (*∗∗*) and (*∗*) are significantly different from the control at p < 0.01 and p < 0.05, respectively.

The antimigration effects of the five potent extracts that inhibited MCF7 cell proliferation were investigated using the wound healing assay.  Figure 1 shows microscope images of the treated cells. Treatment with the plant extracts decreased the migration rate of MCF7 cells in a dose-dependent manner. DMME-treated MCF7 cells showed the lowest percent of cell regrowth; thus it had the highest antimigration action; GSAE was the second most effective extract (Figure 2).

### 3.4. Cell Cycle Analysis by Flow Cytometry

**Figure 3:**
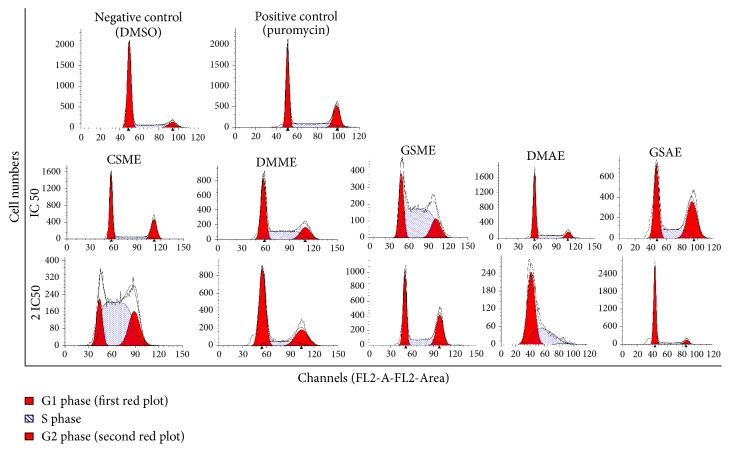
*Effect of the plant extracts on MCF7 cell cycle progression.* The effect of the plant extracts on the cell cycle progression was determined by flow cytometry analysis.* Detarium microcarpum *methanol extract (DMME),* Cassia siamea *methanol extract (CSME),* Guiera senegalensis *methanol extract (GSME),* Detarium microcarpum *aqueous extract (DMAE), and* Guiera senegalensis *aqueous extract (GSAE).

**Figure 4:**
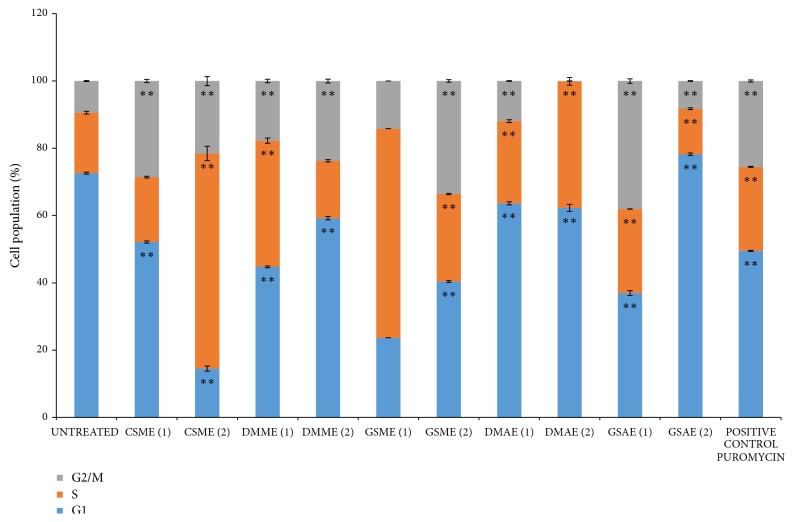
*Population of MCF7 cells at different stages of the cell cycle before and after treatment with plant extracts.* The effect of the plant extracts on apoptosis of MCF7 cells was determined by flow cytometry analysis using Annexin V/PI stain.* Detarium microcarpum *methanol extract (DMME),* Cassia siamea *methanol extract (CSME),* Guiera senegalensis *methanol extract (GSME),* Detarium microcarpum *aqueous extract (DMAE), and* Guiera senegalensis *aqueous extract (GSAE). Values that are marked with (*∗∗*) are significantly different from the control (cell cycle distribution of the untreated cells) at p < 0.01.

The ability of the plant extracts to induce cell cycle arrest was investigated using flow cytometry analysis, and the results are shown in Figure 3. The rate of the arrest induced by some of the plant extracts at a certain phase increased as their concentrations increased, whereas other extracts were more effective at lower doses (Figure 4). For control cells treated with vehicle (0.06% DMSO), 73% of the cell population was in the G1 phase, which meant that the cells were very viable [18]. In contrast, 18% and 9% of the population were in the S and G2/M phases, respectively. All of the potent extracts (CSME, DMAE, DMME, GSME, and GSAE) increased the percentage of the MCF7 cell population that was in the S and G2/M phases, which indicated arrest at these phases (Figure 4). Hence, these extracts induced cell cycle arrest in MCF7 cells (Figure 3).

### 3.5. Analysis of Apoptosis by Flow Cytometry

**Figure 5:**
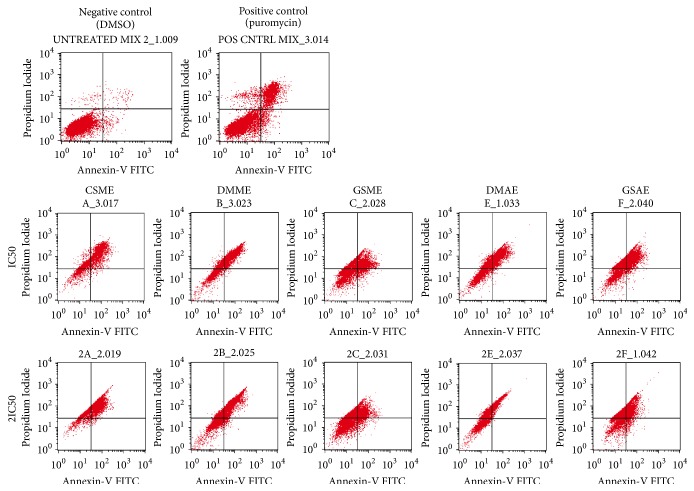
*MCF7 cell population at different stages of apoptosis before and after treatment with plant extracts. Detarium microcarpum *methanol extract (DMME),* Cassia siamea *methanol extract (CSME),* Guiera senegalensis *methanol extract (GSME),* Detarium microcarpum *aqueous extract (DMAE), and* Guiera senegalensis *aqueous extract (GSAE).

**Figure 6:**
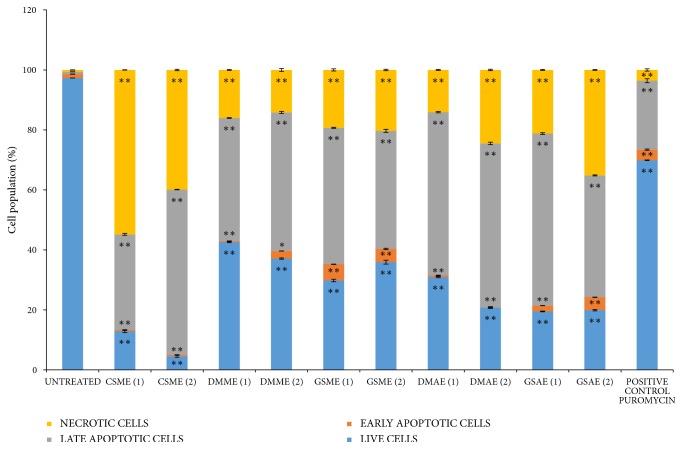
*The cell population at different stages of apoptosis before and after treatment with the plant extracts. Detarium microcarpum *methanol extract (DMME),* Cassia siamea *methanol extract (CSME),* Guiera senegalensis *methanol extract (GSME),* Detarium microcarpum *aqueous extract (DMAE), and* Guiera senegalensis *aqueous extract (GSAE). Values that are marked with (*∗∗*) and (*∗*) are significantly different from the control at p < 0.01 and p < 0.05, respectively.

Apoptosis analysis was carried out using Annexin V/ PI dyes, and Figures 5 and 6 show the results. In the experiment, 97% of the control cells were live and actively viable, which means that the concentration of DMSO (0.06%) used as vehicle did not affect cell proliferation. Puromycin, which was the positive control, induced apoptosis in about 27% of the cells (Figure 6). The percentage of live cells in the cell population that were treated with the specified concentrations (IC_50_, 2IC_50_) of CSME, DMME, DMAE, GSME, and GSAE decreased to (12.8 %, 4.5%), (42.6 %, 37.1 %), (30.9 %, 20.8 %), (29.8 %, 35.9 %), and (19.5 %, 19.9 %), respectively. This was accompanied by increase in the apoptotic cell population at early and late stages but the plant extracts prominently induced late apoptosis. The percentage of late apoptotic cells (0.75 %) in the untreated cell population increased when the cells were treated with the plants extracts. The percentages of late apoptotic cells in the cell population treated with the specified concentrations (IC_50_, 2IC_50_) of CSME, DMME, DMAE, GSME, and GSAE are (31.9 %, 55.1 %), (41. %, 46.2 %), (54.4 %, 54.7 %), (45.5 %, 39.4 %), and (57.4 %, 40.6 %), respectively (Figure 6). In general, CSME, DMME, and DMAE induced late apoptosis in MCF7 cells in a dose-dependent manner, while GSME and GSAE induced late apoptosis in a non-dose-dependent manner.

There was also significant increase in the necrotic cell percentage (0.62 %) of the untreated cells after the cells were treated with the plant extracts. The percentage cell population that were treated with the specified concentrations (IC_50_, 2IC_50_) of CSME, DMME, DMAE, GSME, and GSAE which became necrotic were (54.8%, 39.9 %), (16.0 %, 14.2 %), (14.1 %, 24.5 %), (19.3 %, 20.3 %), and (21.2 %, 35.1 %), respectively (Figure 6). Therefore, the plant extracts induced necrotic cell death of MCF7 cells.

### 3.6. LC/TOF/MS

The probable metabolites of the medicinal plants used in this research were profiled by LC/TOF MS analysis. Thirty-two chemical compounds were identified, and the majority were phenols (Table 2). Five compounds were identified in CSME, 4 in DMME, 8 in GSME, 13 in DMAE, and 12 in GSAE. Some of the compounds were identified in more than one plant, including isochamanetin, phloionolic acid, and gallic acid.

## 4. Discussion

Herbs are major natural sources of antioxidants, which consist mostly of natural phenolic compounds. Many of these compounds have been reported to have antiproliferative effect on cancer cells [19]. In this study, antioxidant and antiproliferative activities of three Nigerian medicinal plants (*D. microcarpum*,* C. siamea*, and* G. senegalensis*) were investigated.

Antioxidants are free radical scavengers and they are known to have inhibitory effects on cancer growth [15, 19]. The DPPH and ABTS test results showed that all six extracts had significant antioxidant capacities (supplementary data) when compared with extracts of other plants. The IC_50_ values of the extracts of* D. microcarpum *and* G. senegalensis *were 13–19 *μ*g/ml according to the DPPH test, and these values are lower than those of other plant samples, such as the methanol extract of* Scurrula ferruginea *stem (27.81 *μ*g/ml), leaf (40.29 *μ*g/ml), and flower (33.35 *μ*g/ml) as estimated by the same antioxidant test [20]. Similarly, the IC_50_ values of the ethanol extract of* Solanum guaraniticum *leaves, the methanol extract of* Mentha pulegium *leaves, and the methanol extract of* Phlomis lanata *leaves were 31.43, 13.5, and 23.9 *μ*g/ml, respectively, based on the DPPH antioxidant assay [21, 22]. The IC_50_ values of the aqueous (464 *μ*g/ml) and methanol (232 *μ*g/ml) extracts of* C. siamea *as estimated by DPPH assay were higher, however, and the range is comparable with the IC_50_ values of other plants, including the methanol extracts of leaves of* Tribulus terrestris *(650 *μ*g/ml),* Bacopa monnieri *(730 *μ*g/ml), and* Trigonella foenum *(810 *μ*g/ml) and the ethanol extract of the aerial part of* Coronopus didymus *(780 *μ*g/ml) [23, 24].

Based on ABTS assay results, the IC_50_ values of the extracts of* D. microcarpum *and* G. senegalensis *ranged from 8 to 13 *μ*g/ml, which were lower than the IC_50_ values of the absolute ethanol (70 *μ*g/ml) and water (390 *μ*g/ml) extracts of* Angelica sinensis *root [25] and the methanol (70 *μ*g/ml) and water (310 *μ*g/ml) extracts of the whole plant of* Atriplex laciniata *[26]. The IC_50_ values of the aqueous and methanol extracts of* C. siamea *were 115 and 160 *μ*g/ml, respectively, which shows that this plant also has substantial antioxidant capacity. Together, the results of both assays suggest that the extracts of* D. microcarpum*,* C. siamea*, and* G. senegalensis *possess substantial antioxidant capacities [27].

Because breast cancer remains one of the most common cancer types, especially among women, the potential of the plants as possible sources of oestrogen responsive breast cancer chemotherapy was preliminarily examined* in vitro *using MCF7 breast cancer cell as a model. The cell viability assay results confirmed the antiproliferative effects of the plant extracts on MCF7 growth, except for CSAE, and the highest inhibitory effect was exhibited by DMAE (Table 1). The inhibitory effects of the plant extracts could be due to their significant antioxidant capacities, whereas the opposite may be true for CSAE, which had the lowest antioxidant activity. Antioxidants of plant origin or other natural sources have been shown to cause death by induction of apoptosis and cycle arrest in breast, lung, colorectal, and alveolar cancers, among others [27–29]. In general, the trend of MCF7 cell inhibition by the plant extracts is in agreement with that of their respective antioxidant capacities.

In order to identify the cellular processes that were modulated by the plant extracts to inhibit cancer cell proliferation, the effects of the extracts on metastasis, apoptosis, and cell cycle progression were investigated. A potential anticancer agent should have a negative effect on the rapid migration of cancer cells to inhibit or decrease the metastasis rate of the tumour cells [30]. The wound healing assay revealed the plant extracts had an antimigration effect on MCF7 cell proliferation (Figures 1 and 2). Thus, the five potent plant extracts not only retarded MCF7 cell growth but they can also localize cell growth by decreasing the rate of cell metastasis. Apoptosis is a programmed cell death process that is usually used by malfunctioning cells to induce self-death [28]. Therefore, activation of the process by an agent results in inhibition of proliferation and death of the cancer cells. The five potent extracts activated apoptosis in MCF7 cells in a dose dependent manner (Figures 5 and 6). The apoptosis results are similar to other reports of the apoptotic effect of herbal products [28, 31, 32]. The plant extracts also induced cell cycle arrest at the S phase in MCF7 cells, which indicated that they caused DNA damage and irregularity in genome replication of the cells. Some of the cancer cells that escaped S phase arrest were not able to segregate and divide properly, and they accumulated in the G2/M phase. Arrest at this stage would eventually lead to cell death because the DNA damage could not be repaired [33].

The LC/TOF/MS chemical profiling results revealed that all of the plant extracts contained phenolic compounds, and some contained omega-6 fatty acid in addition to phenols (Table 2). These compounds are antioxidants because they are electron-rich compounds, and they are capable of donating electrons to scavenge free radicals [15, 34]. Therefore, these results supported the DPPH and ABTS results, which showed that the plant extracts possess antioxidant capacities. These compounds could be responsible for the antiproliferative, apoptotic, and cell cycle arrest effects of the extracts on MCF7 breast cancer cells, because phenolics and omega-6 fatty acids have been widely reported to have inhibitory effects on cancer cells [19, 27, 34]. Specifically, isorhamnetin [35, 36] in GSAE and GSME; eupatorin [37] in GSAE; alpinumisoflavone [38], procyanidin B3 [39], and syringin [40] in DMAE; gallic acid [41, 42] in DMAE and GSAE; and serpyllin [43] in CSME and DMAE have all been reported to have antiproliferative effects on cancer cells.

## 5. Conclusion

Based on* in vitro* analysis, the extracts of stem bark from three Nigerian medicinal plants (*D. microcarpum*,* G. senegalensis, *and* C. siamea*) have significant antioxidant capacities as determined by ABTS and DPPH assays, which are due to their phenolic compound constituents as identified by LCMS analysis. The methanol and aqueous extracts from the stem barks of these plants inhibited the proliferation of MCF7 breast cancer cells except the aqueous extract of* C. simamea*. The potent plant extracts also induced cell cycle arrest and apoptosis and inhibited metastasis in MCF7 breast cancer cells. In general, it could be said that the plants' extracts are potential sources of antibreast cancer agents.

## Figures and Tables

**Table 1:** * Cell viability assay.* The viability of MCF7 cells was determined using PrestoBlue dye following 72-hour treatment with the plant extracts. *Detarium microcarpum *methanol extract (DMME), *Cassia siamea *methanol extract (CSME), *Guiera senegalensis *methanol extract (GSME), *Detarium microcarpum *aqueous extract (DMAE), *Cassia siamea *aqueous extract (CSAE), and *Guiera senegalensis *aqueous extract (GSAE).

Concentration (*µ*g/ml)	Percentage cell viability					
	DMME	DMAE	GSME	GSAE	CSME	CSAE
15.625	92.87 ± 8.10	121.96 ± 6.01	101.78 ± 2.41	119.75 ±1.99	80.27 ± 6.39	113.12 ± 2.17
31.25	90.41 ± 14.32	105.11 ± 0.00	107.25 ± 6.02	88.54 ± 8.54	82.83 ± 7.79	117.40 ± 1.27
62.5	80.04 ± 13.51	59.94 ± 0.00	68.78 ± 3.23	91.02 ± 7.32	100.11 ± 4.02	122.10 ± 1.13
125	51.51 ± 2.68	33.98 ± 0.00	64.10 ± 0.00	54.97 ± 3.09	51.28 ± 4.02	116.44 ± 0.73
250	29.10 ± 0.10	22.51± 4.71	38.13 ± 5.04	32.18 ± 0.00	36.34 ± 4.57	119.34 ± 1.70
500	34.26 ± 3.97	33.01 ± 3.55	25.75 ± 1.26	36.33 ± 0.00	13.77 ± 5.18	93.51 ± 14.17

**Table 2:** The probable compounds found in the plant extracts as identified by LC/TOF/MS.

Number	Retention time (min)	Molecular formula	Experimental m/z	Calculated m/z	Error (ppm)	Proposed compound	Medicinal plant
1	1.975	C20H20O8	388.1158	387.1080	-6.2	Serpyllin	CSME, DMAE

2	9.363	C27H30O13	562.1686	561.1608	-44.2	6,8-Di-c-rhamnosylapigenin	DMAE

3	10.531	C21H30O10	442.1839	441.1761	-86.6	Lusitanicoside	CSME

4	13.520	C18H36O5	332.2563	331.2484	-84.2	Phloionolic acid	CSME, DMAE, & GSAE

5	17.573	C24H40O2	360.3028	359.2950	-113.0	Tetracosatetraenoic acid n-6	CSME, DMAE, & GSAE

6	24.463	C15H22O4	266.1518	265.1440	0.8	Cumanin	CSME, GSM

7	1.946	C22H28O6	388.1886	387.1808	-104.4	Cyclomammein	DMM

8	2.160	C24H24O5	392.1624	391.1545	-38.6	Calabaxanthone	DMM

9	7.294	C22H18O7	394.1053	393.0974	-47.3	Justicidin A	DMM

10	8.789	C20H20O8	388.1158	387.1080	-98.9	Demethylnobiletin	DMM

11	1.952	C22H18O6	378.1103	377.1025		Jamaicin	GSM

12	3.773	C21H22O4	338.1518	337.1346	-98.8	Gancaonin X	GSM

13	7.398	C22H18O5	362.1154	361.1076	-43.2	Isorhamnetin	GSAE, GSM

14	8.609	C35H28O22	800.1072	799.1029	-0.4	1,3,4,5-Tetra-O-galloylquinic acid	GSAE, GSM

15	8.965	C30H16O8	504.0845	503.0767	6.2	Hypericin	GSM

16	18.351	C37H60O12	696.4085	695.4007	-1.3	Glucosyl passiflorate	GSM

17	2.320	C18H18O7	346.1053	345.0974	-57.1	Amorphaquinone	DMAE

18	3.145	C7H6O5	170.0215	169.0137	-21.9	Gallic acid	DMAE, GSAE

19	5.958	C27H30O14	578.1636	577.1557	-39.3	Violanthin	DMAE

20	7.272	C17H24O9	372.1420	371.1342	-107.8	Syringin	DMAE

21	7.414	C20H16O5	336.0998	335.0919	-52.5	Isoderrone	DMAE

22	8.025	C30H26O12	578.1424	577.1346	-1.0	Procyanidin B3	DMAE

23	8.767	C20H16O5	336.0998	335.0919	-53.7	Alpinumisoflavone	DMAE

24	9.803	C30H26O11	562.1475	561.1397	-8.0	Epifisetinidol-(4beta->8)-catechin	DMAE

25	24.437	C26H44O2	388.3341	387.3263	-104.3	3-Hydroxy-1-phenyl-1-eicosanone	DMAE

26	2.589	C18H16O7	344.0896	343.0818	-42.6	Eupatorin	GSAE

27	4.240	C21H20O14	496.0853	495.0775	-7.5	3,4-Di-O-galloylquinic acid	GSAE

28	4.582	C18H10O6	322.0477	321.0399	-53.9	7,7-Dihydroxy-6,8′-bicoumarin	GSAE

29	5.436	C7H6O4	154.0266	153.0188	-30.7	2,3-Dihydroxybenzoic acid	GSAE

30	7.042	C22H20O10	444.1056	443.0978	-82.6	Rothindin	GSAE

31	10.929	C24H24O11	488.1319	487.1240	-73.3	Trifolirhizin-6′-monoacetate	GSAE

32	24.460	C26H44O2	388.3341	387.3263	-106.4	3-Hydroxy-1-phenylicosan-1-one	GSAE

*Detarium microcarpum *methanol extract (DMME), *Cassia siamea *methanol extract (CSME), *Guiera senegalensis *methanol extract (GSME), *Detarium microcarpum *aqueous extract (DMAE), and *Guiera senegalensis *aqueous extract (GSAE)

## Data Availability

The data used to support the findings of this study are available from the corresponding author upon request.

## Supplementary Materials

Supplementary Materials SUPPLEMENTARY DATA - ANTIOXIDANT STANDARD CURVES AND MS CHROMATOGRAMS. Figure S1.* ABTS antioxidant capacities of the plant extracts*.* Detarium microcarpum* methanol extract (DMME),* Cassia siamea* methanol extract (CSME),* Guiera senegalensis* methanol extract (GSME),* Detarium microcarpum* aqueous extract (DMAE),* Cassia siamea* aqueous extract (CSAE), and* Guiera senegalensis* aqueous extract (GSAE). Values that are marked with (*∗∗*) are significantly different from the blank/control at p < 0.01. Figure S2.* DPPH antioxidant capacities of the plant extracts*.* Detarium microcarpum* methanol extract (DMME),* Cassia siamea* methanol extract (CSME),* Guiera senegalensis* methanol extract (GSME),* Detarium microcarpum* aqueous extract (DMAE),* Cassia siamea* aqueous extract (CSAE), and* Guiera senegalensis* aqueous extract (GSAE). Values that are marked with (*∗∗*) are significantly different from the blank/control at p < 0.01. Figure S3. MS chromatogram of* Cassia siamea* methanol extract (CSME). The compounds attributed to the numbered peaks and their respective properties as identified using METLIN are in Table 2. Figure S4. MS chromatogram of* Detarium microcarpum* aqueous extract (DMAE). The compounds attributed to the numbered peaks and their respective properties as identified using METLIN are in Table 2. Figure S5. MS chromatogram of* Detarium microcarpum* methanol extract (DMME). The compounds attributed to the numbered peaks and their respective properties as identified using METLIN are in Table 2. Figure S6. MS chromatogram of* Guiera senegalensis* aqueous extract (GSAE). The compounds attributed to the numbered peaks and their respective properties as identified using METLIN are in Table 2. Figure S7. MS chromatogram of* Guiera senegalensis* methanol extract (GSME). The compounds attributed to the numbered peaks and their respective properties as identified using METLIN are in Table 2.
