# Corrigendum to “*Detarium microcarpum*, *Guiera senegalensis*, and *Cassia siamea* Induce Apoptosis and Cell Cycle Arrest and Inhibit Metastasis on MCF7 Breast Cancer Cells”

**DOI:** 10.1155/2019/1529570

**Published:** 2019-09-08

**Authors:** Ismail Abiola Adebayo, Haladu Ali Gagman, Wasiu Gbolahan Balogun, Mowaffaq Adam Ahmed Adam, Rafedah Abas, Khalid Rehman Hakeem, Nik Ahmad Irwan Izzauddin Bin Nik Him, Muhammad Razip Bin Samian, Hasni Arsad

**Affiliations:** ^1^Integrative Medicine Cluster, Advanced Medical and Dental Institute, Universiti Sains Malaysia, Bertam, 13200 Kepala Batas, Pulau Pinang, Malaysia; ^2^School of Biological Sciences, Universiti Sains Malaysia, George Town, 11800 Penang, Malaysia; ^3^Department of Biological Sciences, Faculty of Sciences, Bauchi State University Gadau, 751 Itas Gadau, Nigeria; ^4^Infectomics Cluster, Advanced Medical and Dental Institute, Universiti Sains Malaysia, George Town, Penang, Malaysia; ^5^Centralized Research Labs (CRL), Advanced Medical and Dental Institute USM, Bertam, 13200 Kepala Batas, Penang, Malaysia; ^6^Department of Biological Science, Faculty of Science, King Abdulaziz University, P.O. Box 80203, Jeddah, Saudi Arabia

In the article titled “*Detarium microcarpum*, *Guiera senegalensis*, and *Cassia siamea* Induce Apoptosis and Cell Cycle Arrest and Inhibit Metastasis on MCF7 Breast Cancer Cells” [1], an acknowledgment should be added as follows:

The article is partially funded by USM-Bridging Grant: (304.CIPPT.6316239).
