# Supplementary material for: Detarium microcarpum, Guiera senegalensis, and Cassia siamea Induce Apoptosis and Cell Cycle Arrest and Inhibit Metastasis on MCF7 Breast Cancer Cells
Source: Evid Based Complement Alternat Med. 2019 May 23;2019:6104574. doi: 10.1155/2019/6104574 (PMC6556270; doi:10.1155/2019/6104574)
Supplement: Supplementary Materials — SUPPLEMENTARY DATA - ANTIOXIDANT STANDARD CURVES AND MS CHROMATOGRAMS. Figure S1. ABTS antioxidant capacities of the plant extracts. Detarium microcarpum methanol extract (DMME), Cassia siamea methanol extract (CSME), Guiera senegalensis methanol extract (GSME), Detarium microcarpum aqueous extract (DMAE), Cassia siamea aqueous extract (CSAE), and Guiera senegalensis aqueous extract (GSAE). Values that are marked with (∗∗) are significantly different from the blank/control at p < 0.01. Figure S2. DPPH antioxidant capacities of the plant extracts. Detarium microcarpum methanol extract (DMME), Cassia siamea methanol extract (CSME), Guiera senegalensis methanol extract (GSME), Detarium microcarpum aqueous extract (DMAE), Cassia siamea aqueous extract (CSAE), and Guiera senegalensis aqueous extract (GSAE). Values that are marked with (∗∗) are significantly different from the blank/control at p < 0.01. Figure S3. MS chromatogram of Cassia siamea methanol extract (CSME). The compounds attributed to the numbered peaks and their respective properties as identified using METLIN are in Table 2. Figure S4. MS chromatogram of Detarium microcarpum aqueous extract (DMAE). The compounds attributed to the numbered peaks and their respective properties as identified using METLIN are in Table 2. Figure S5. MS chromatogram of Detarium microcarpum methanol extract (DMME). The compounds attributed to the numbered peaks and their respective properties as identified using METLIN are in Table 2. Figure S6. MS chromatogram of Guiera senegalensis aqueous extract (GSAE). The compounds attributed to the numbered peaks and their respective properties as identified using METLIN are in Table 2. Figure S7. MS chromatogram of Guiera senegalensis methanol extract (GSME). The compounds attributed to the numbered peaks and their respective properties as identified using METLIN are in Table 2. [file 6104574.f1.docx]

**SUPPLEMENTARY DATA - ANTIOXIDANT STANDARD CURVES AND MS CHROMATOGRAMS**


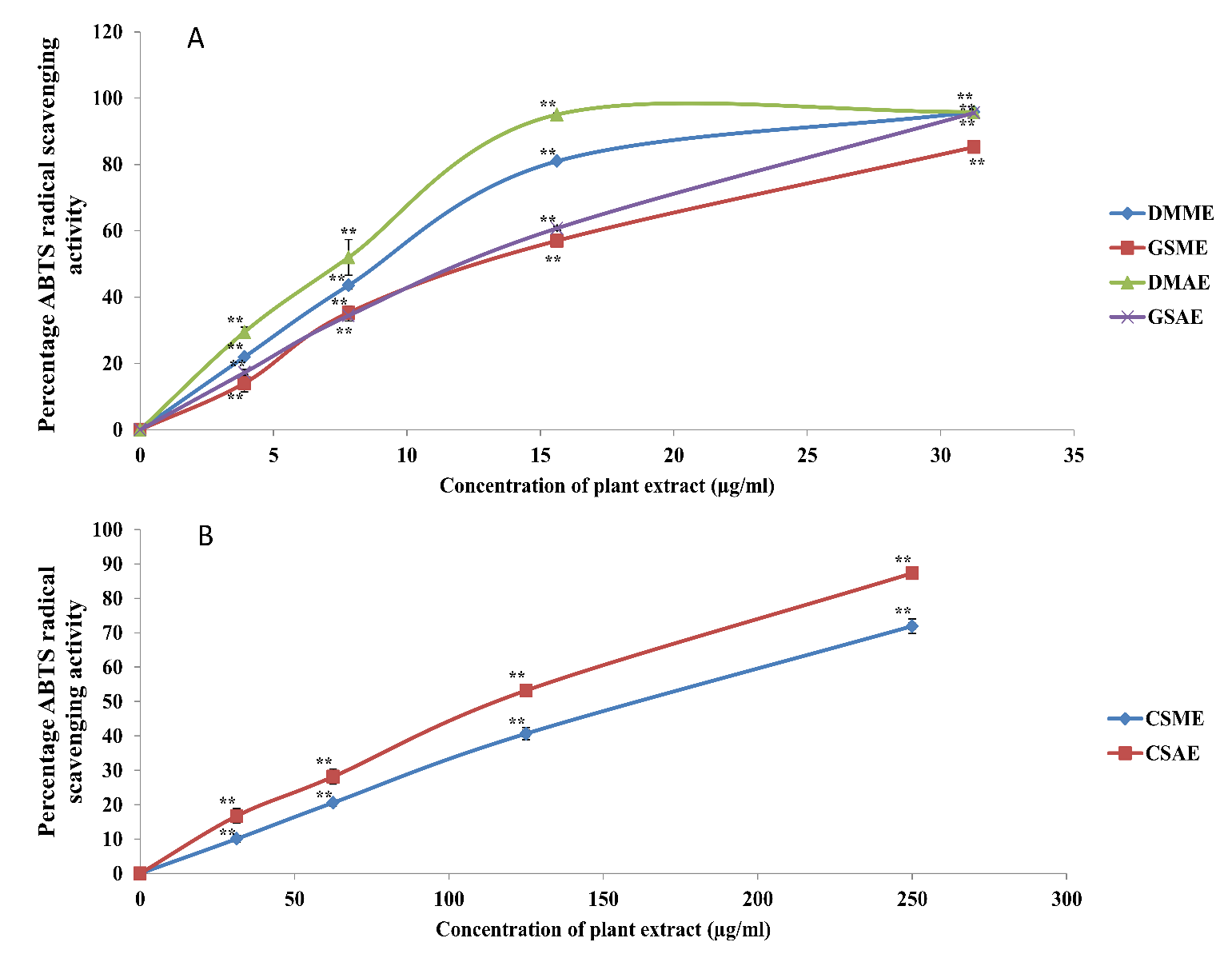


**Figure S1. ABTS antioxidant capacities of the plant extracts.** *Detarium microcarpum* methanol extract (DMME), *Cassia siamea* methanol extract (CSME), *Guiera senegalensis* methanol extract (GSME), *Detarium microcarpum* aqueous extract (DMAE), *Cassia siamea* aqueous extract (CSAE), and *Guiera senegalensis* aqueous extract (GSAE). Values that are marked with (**) are significantly different from the blank/control at p < 0.01.


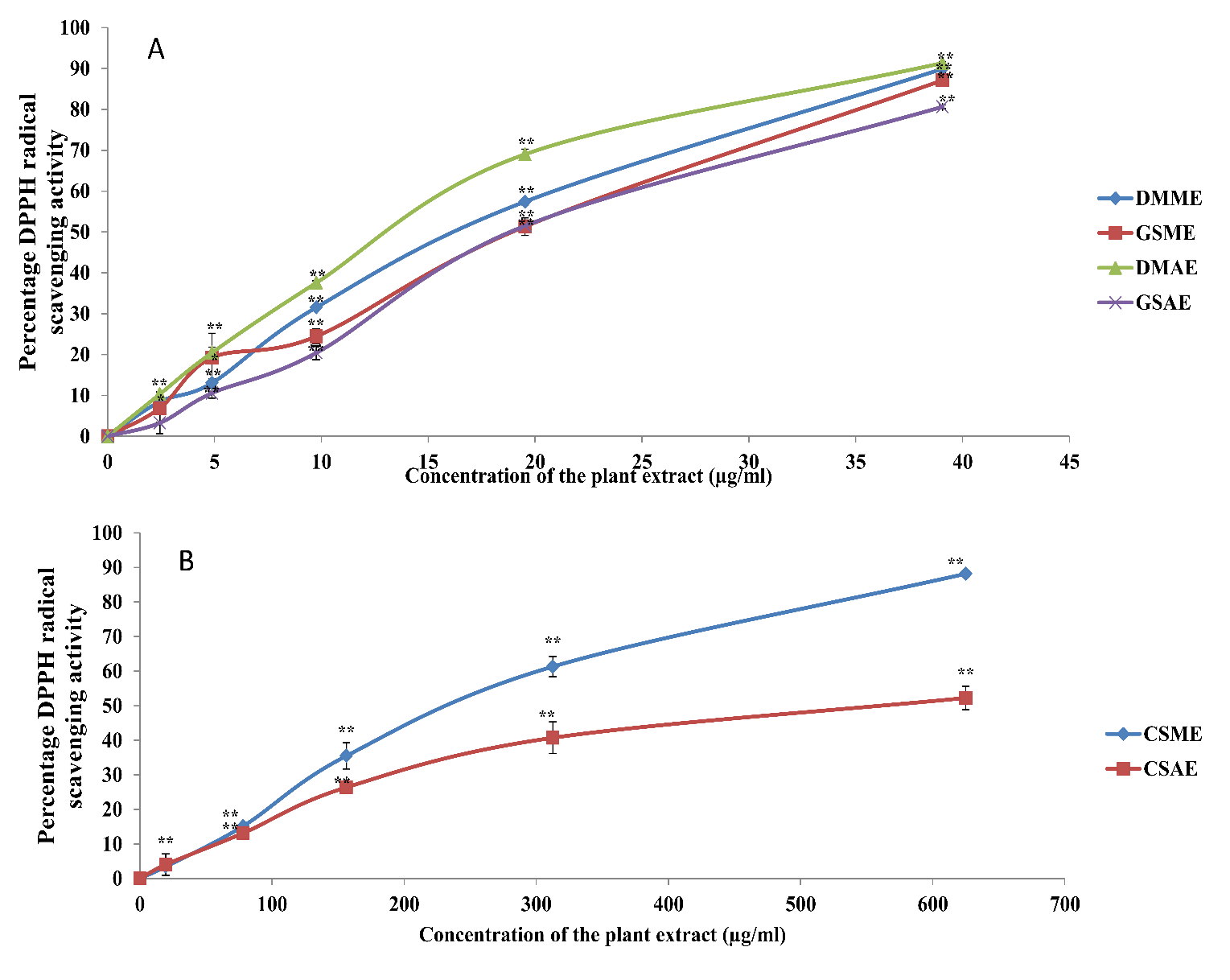


**Figure S2. DPPH antioxidant capacities of the plant extracts.** *Detarium microcarpum* methanol extract (DMME), *Cassia siamea* methanol extract (CSME), *Guiera senegalensis* methanol extract (GSME), *Detarium microcarpum* aqueous extract (DMAE), *Cassia siamea* aqueous extract (CSAE), and *Guiera senegalensis* aqueous extract (GSAE). Values that are marked with (**) are significantly different from the blank/control at p < 0.01.


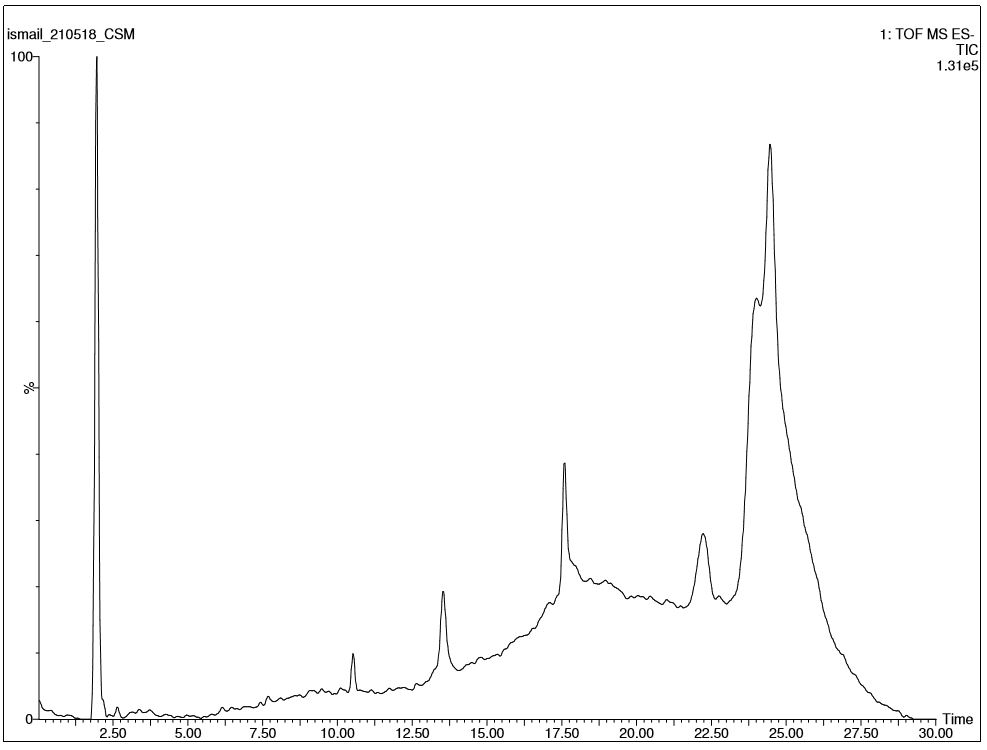


1

4

3

5

6

Figure S3. MS chromatogram of *Cassia siamea* methanol extract (CSME). The compounds attributed to the numbered peaks and their respective properties as identified using METLIN are in Table 2


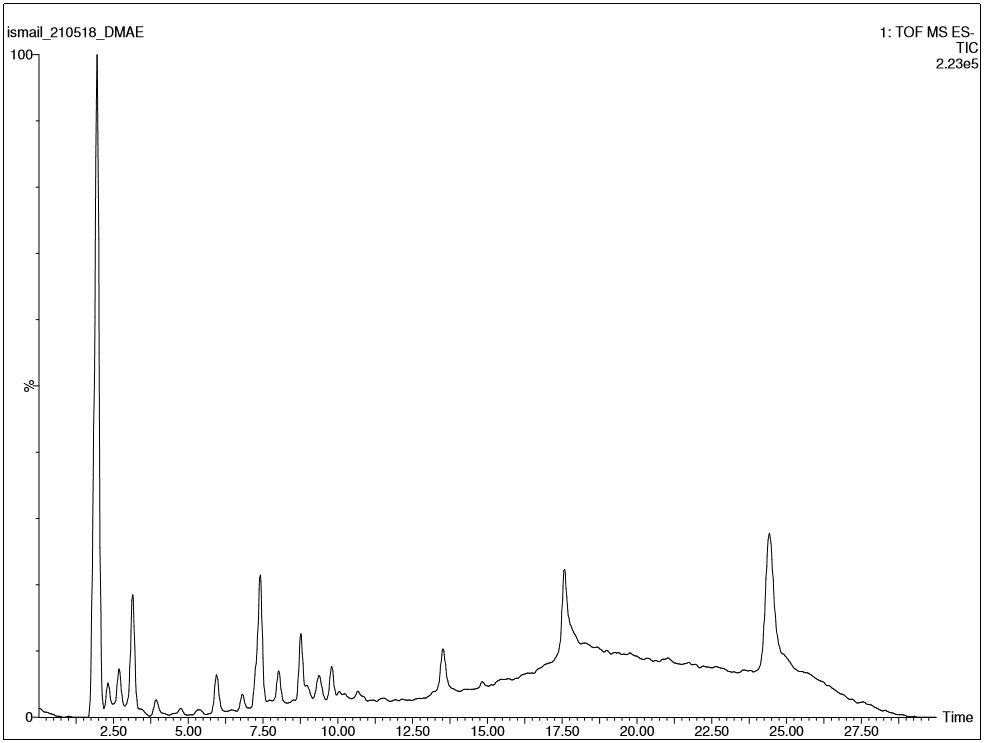


2

4

5

17

18

21

1

19

23

24

22

25

Figure S4. MS chromatogram of *Detarium microcarpum* aqueous extract (DMAE). The compounds attributed to the numbered peaks and their respective properties as identified using METLIN are in Table 2


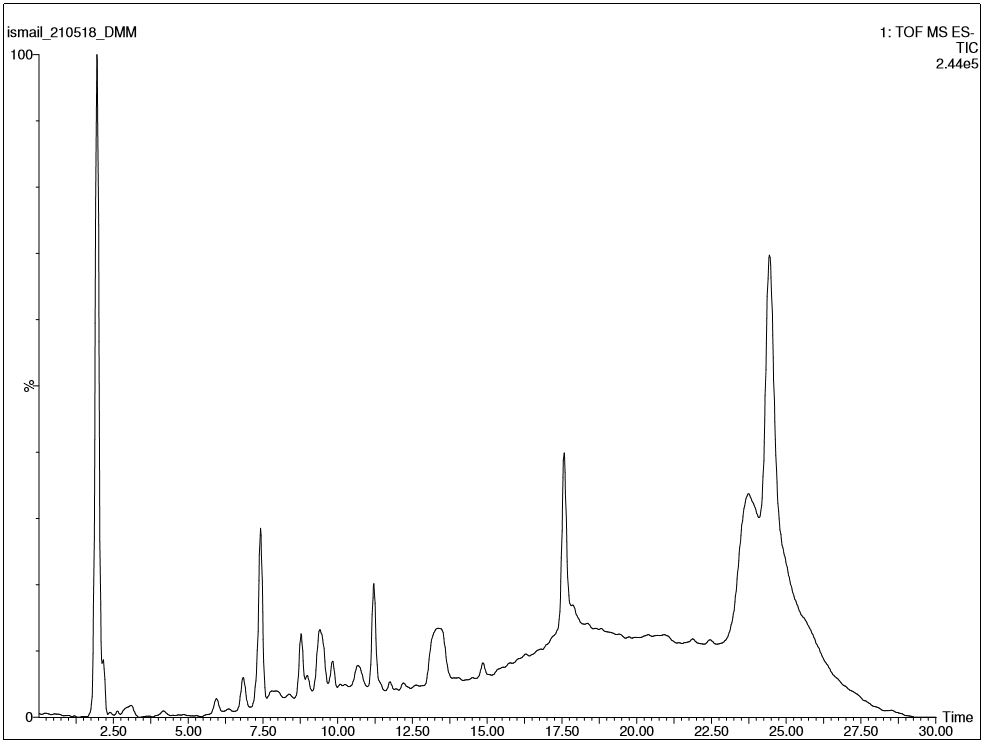


7

8

10

9

Figure S5. MS chromatogram of *Detarium microcarpum* methanol extract (DMME). The compounds attributed to the numbered peaks and their respective properties as identified using METLIN are in Table 2


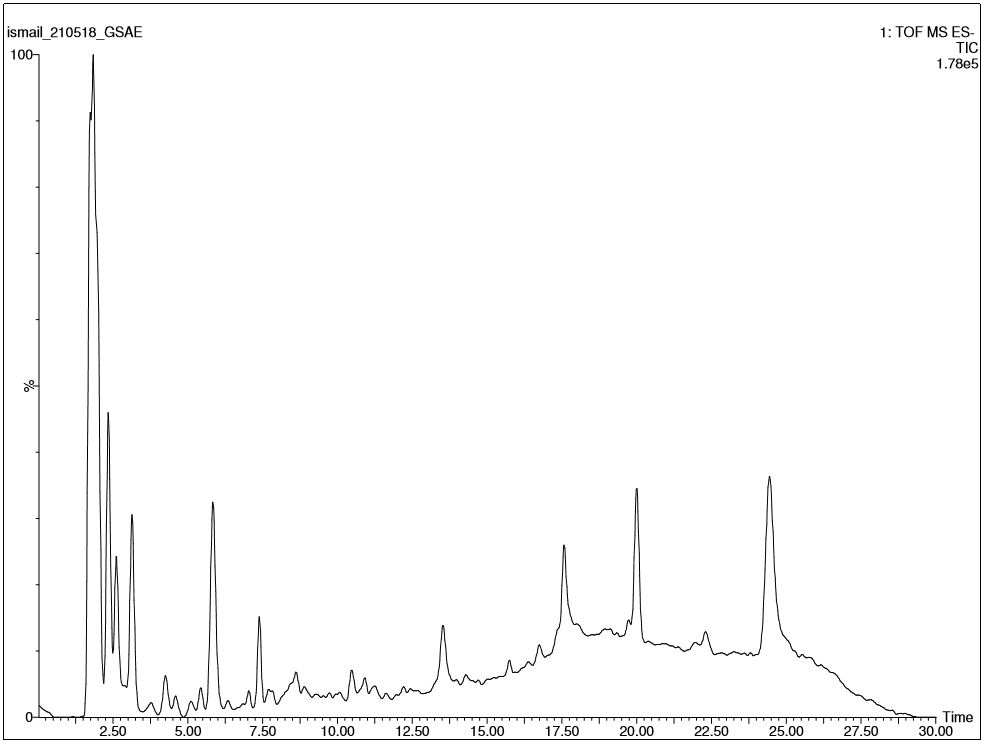


4

5

13

18

14

26

27

28

29

30

31

32

Figure S6. MS chromatogram of *Guiera senegalensis* aqueous extract (GSAE). The compounds attributed to the numbered peaks and their respective properties as identified using METLIN are in Table 2


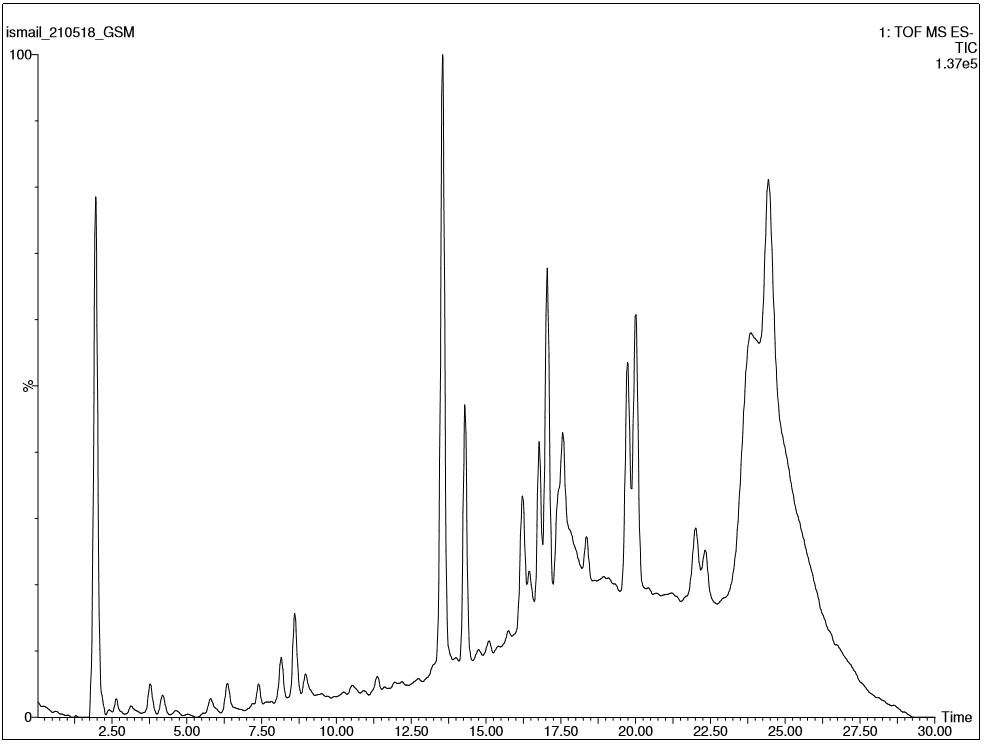


6

16

15

13

14

12

11

Figure S7. MS chromatogram of *Guiera senegalensis* methanol extract (GSME). The compounds attributed to the numbered peaks and their respective properties as identified using METLIN are in Table 2
